# Advancements in Fatigue Detection: Integrating fNIRS and Non-Voluntary Attention Brain Function Experiments

**DOI:** 10.3390/s24103175

**Published:** 2024-05-16

**Authors:** Ting Li, Peishuai Liu, Yuan Gao, Xiang Ji, Yu Lin

**Affiliations:** 1 Institute of Biomedical Engineering, Chinese Academy Medical Sciences & Peking Union Medical College, Tianjin 300192, China; lps2019@tju.edu.cn (P.L.); jixiang8394@163.com (X.J.); 2 Institute of Integrated Circuit Science and Engineering, University of Electronical Science and Technology of China, Chengdu 611731, China; q823410144@163.com; 3 North Carolina State University, Raleigh, NC 27695, USA; linyu61327@163.com

**Keywords:** involuntary attention, fatigue driving, fNIRS, behavioral monitoring

## Abstract

Background: Driving fatigue is a significant concern in contemporary society, contributing to a considerable number of traffic accidents annually. This study explores novel methods for fatigue detection, aiming to enhance driving safety. Methods: This study utilizes electroencephalography (EEG) and functional near-infrared spectroscopy (fNIRS) to monitor driver fatigue during simulated driving experiments lasting up to 7 h. Results: Analysis reveals a significant correlation between behavioral data and hemodynamic changes in the prefrontal lobe, particularly around the 4 h mark, indicating a critical period for driver performance decline. Despite a small participant cohort, the study’s outcomes align closely with established fatigue standards for drivers. Conclusions: By integrating fNIRS into non-voluntary attention brain function experiments, this research demonstrates promising efficacy in accurately detecting driving fatigue. These findings offer insights into fatigue dynamics and have implications for shaping effective safety measures and policies in various industrial settings.

## 1. Introduction

In contemporary times, the proliferation of motor vehicles has escalated concerns, elevating driving safety to a societal problem. The World Health Organization (WHO) somberly reports an annual toll of 1.3 million lives succumbing to traffic accidents, with driving fatigue emerging as a prominent contributory factor [1]. As the number of motor vehicles surges, the imperative to ensure driving safety intensifies [2]. The rise in traffic accidents, as highlighted by the WHO, underscores the urgency of addressing driving-related challenges, with driving fatigue at the forefront. The surge in property losses due to driver fatigue was exemplified by Zhang et al.’s findings in China, where 9.26% of traffic accidents in China alone were caused by driver fatigue, resulting in property losses exceeding USD 5.28 million [2].

Driving fatigue refers to the impact of physiological or psychological fatigue during the driving process, leading to a decline in driving skills [3]. Detecting fatigue during driving presents a complex challenge, distinct from established methods for testing intoxicated driving through blood alcohol concentration [4,5]. In the initial stages of detecting driver fatigue, researchers commonly rely on the dynamic interaction between drivers and vehicles [6,7], encompassing factors like steering wheel grasp intensity, steering frequency, and steering wheel angle [8]. However, these behavioral indicators exhibit limited correlation with physiological conditions and can be notably influenced by the specific vehicle model. With the swift evolution of computer vision technologies, the current trend in fatigue monitoring revolves around assessing head movements, pupil diameter, blink frequency, duration between blinks, the percentage of time with closed eyes surpassing the pupil (PERCLOS), eye aspect ratio, saccades, and various eye movement patterns [9]. Despite this, computer vision analysis is generally viewed as having an indirect connection to physiological states, and its accuracy can be easily impacted by environmental and lighting conditions [10]. Analytical methods based on brain information continue to be hailed as the most direct and effective means of fatigue detection [10]. The fatigue analysis method based on brain information has consistently been regarded as the most direct and effective approach to detect fatigue. Currently, EEG (electroencephalography) and fNIRS (functional near-infrared spectroscopy) are commonly employed methods for fatigue detection [11,12,13,14,15,16,17]. Most researchers are attempting to establish more precise real-time monitoring models by optimizing parameters and algorithms. The longest continuous driving time in experimental setups is 4 h. However, Meng et al. conducted an investigation into the approximate driving time before accidents occur. The results indicate that taxi drivers typically experience fatigue-related accidents after driving for approximately 9.3 h, while truck drivers tend to have fatigue-related accidents after driving for around 8.2 h [18]. This section delves into the evolving landscape of fatigue detection methods, emphasizing the inherent limitations of early approaches rooted in interactive behavior information and computer vision analysis. Apparently, the duration of existing studies may not be sufficient to fully analyze the entire fatigue process. In this paper, the simulated driving experiment was extended to 7 h to explore the changes in drivers’ behavior and the hemodynamics of the prefrontal lobe in a more comprehensive paradigm.

Several studies have presented evidence linking fatigue with visual involuntary attention [19]. Involuntary attention, triggered by external stimuli, is crucial for threat detection and self-preservation [20]. Therefore, studying brain function alterations during visual involuntary attention could unveil new indicators for assessing fatigue during prolonged driving. Many teams have investigated the fatigue status of industrial operators [21], but, at present, questionnaires are mostly used for investigation, which lack correlation with physiological signals. However, fNIRS combined with involuntary attention brain function experiments, proposed by this team, could be a better way for fatigue Monitoring.

To delve deeper into physiological brain changes during fatigue, we employed functional near-infrared spectroscopy (fNIRS) to monitor hemodynamic shifts in the forehead during extended driving. Previous research has explored various physiological signals for fatigue monitoring, including heart-based and eye-based signals [9,22,23,24], yet their correlation with driving fatigue remains weak. Brain-based signals, such as electroencephalography (EEG) and fNIRS, offer direct insights into brain function related to fatigue. While EEG requires participants to wear electrode caps and minimize movements for signal quality, fNIRS offers a less intrusive alternative. Participants only need to wear a flexible film electrode on their forehead for fNIRS data collection.

Despite the modest participant cohort of eight, the study’s outcomes align closely with established fatigue standards for drivers. The paper concludes by highlighting the groundbreaking efficacy demonstrated in combining fNIRS with non-voluntary attention brain function experiments, not only in accurately detecting driving fatigue but also in offering prospective applications in industrial production settings. These findings pave the way for a nuanced understanding of fatigue dynamics, which is imperative for shaping effective safety measures and policies.

## 2. Materials and Methods

### 2.1. Participants

This study recruited 17 healthy right-handed volunteers with valid driver’s licenses; unfortunately, only 11 volunteers (7 males and 4 females) completed the experiment. The average age of the volunteers was 22.0 years (ranging from 21 to 25 years), and their average years of education were 16.1 years (ranging from 15 to 17 years). All participants had no history of sleep disorders, neurological disorders, mental disorders, or substance abuse. Participants had normal vision or corrected-to-normal vision to meet driving requirements. All volunteers signed an informed consent form to participate in the experiment. Three participants (two males, one female) engaged in excessive activities such as drinking water, using the restroom, and eating snacks too much during the data collection process, affecting data quality. Therefore, even though they completed the experiment, their data were excluded from the data analysis. This study was carried out at room temperature with no external light source in the room; only the indoor light source provided lighting. The location did not change during the experiment, and the period from the initial experiment to the final experiment was one month, so there was no significant difference in humidity.

Participants were instructed to carry out their daily activities during the day and get adequate sleep (8 h) the night before the experiment. Participants were not allowed to take naps during the experiment. Additionally, within the 24 h preceding the experiment, participants were restricted from consuming alcohol, tea, nicotine, and caffeine.

### 2.2. Experimental Setup

Presentation, developed by Neurobehavioral Systems Inc. in the United States, is an innovative platform tailored for conducting psychology experiments and delving into cognitive science research. Utilizing Presentation (New Brunswick Scient Inc., Edison, NJ, USA), this study harnessed its capabilities to regulate the visual stimuli in the experiment and focused on involuntary attention brain function, concurrently capturing the visual stimuli and behavioral responses throughout the duration of the experiment. The experimental framework for exploring involuntary attention brain function opted for the traditional visual Oddball paradigm, incorporating both voluntary and involuntary components.

**Figure 1:**
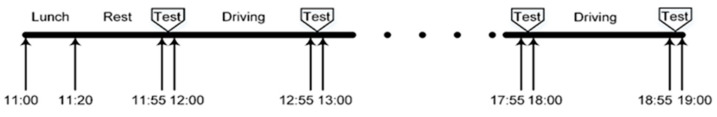
Testing paradigm of visual involuntary attention.

Traffic scenes were utilized as stimuli to replicate the experimental paradigm for traffic signals [25]. The visual stimulation of the traffic road (Figure 1) included braking situations that required deceleration or other adjustments in driving. Safe scenarios presented visuals of traffic situations that could be navigated at the current speed. Sub-scenarios with a low probability were introduced based on the aforementioned situations, featuring either a red light or green light in predetermined proportions, necessitating participants’ judgment. In this experiment, 30 stimuli with a red light, 30 stimuli with a green light, and 140 stimuli for braking and safe scenarios were presented in a pseudo-random manner, ensuring there were no consecutive red or green light scenes during the experiment. Each stimulus was displayed for a duration of 900 to 1100 milliseconds. Throughout the experiment, participants were instructed to engage the left mouse button upon the appearance of a red light, the right mouse button for a green light, and to refrain from any operation during other scenes. Simultaneously, Presentation recorded participants’ responses and reaction time data. All participants adhered to simulated driving instructions within the simulation environment throughout the stimulation process.

This study utilized a driving simulator manufactured by Keteng, equipped with a force feedback steering wheel, brakes, accelerator, clutch, and gear shifter, mirroring those found in conventional automobiles. The DASS simulation software (developed by Stowood Scientific Instruments, Oxford, UK) was employed to project road traffic driving scenarios onto a computer screen. These simulated driving environments encompassed authentic roads, pedestrians, diverse vehicle types, traffic lights, signal signs, and various structures.

**Figure 2:**
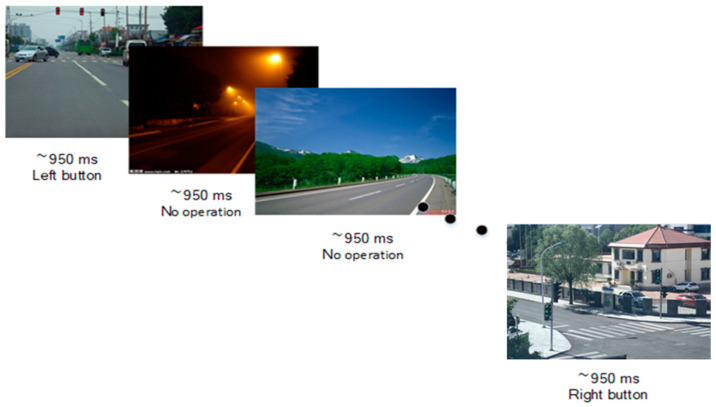
Timeline of the experimental procedure.

Upon arrival at the laboratory at 11:00 a.m. (Figure 2), participants were instructed to have lunch and then rest until the experiment commenced at 11:55 a.m. The duration of the experiment totaled 7 h. The experiment commenced with the involuntary attention paradigm, where participants experienced a completely non-fatigued state for approximately 5 min. Subsequently, the driving simulation was initiated and lasted for about 55 min. Following the completion of the first driving simulation, the non-voluntary brain function experiment was conducted after a brief 5 min interval. The experiment comprised a total of seven simulated driving sessions and eight non-voluntary attention experiments. During the non-voluntary attention brain function experiment, participants’ foreheads were fitted with flexible near-infrared probes to capture hemodynamic signals from the prefrontal cortex. Participant responses and reaction times were automatically recorded based on keyboard inputs. Throughout the non-voluntary attention brain function experiment, participants’ eyes maintained a distance of 120 cm from the screen center, with a vertical viewing angle of 3.0° and a horizontal viewing angle of 3.2°. Participants were instructed to respond promptly and accurately to stimuli requiring a reaction by pressing the designated key.

**Figure 3:**
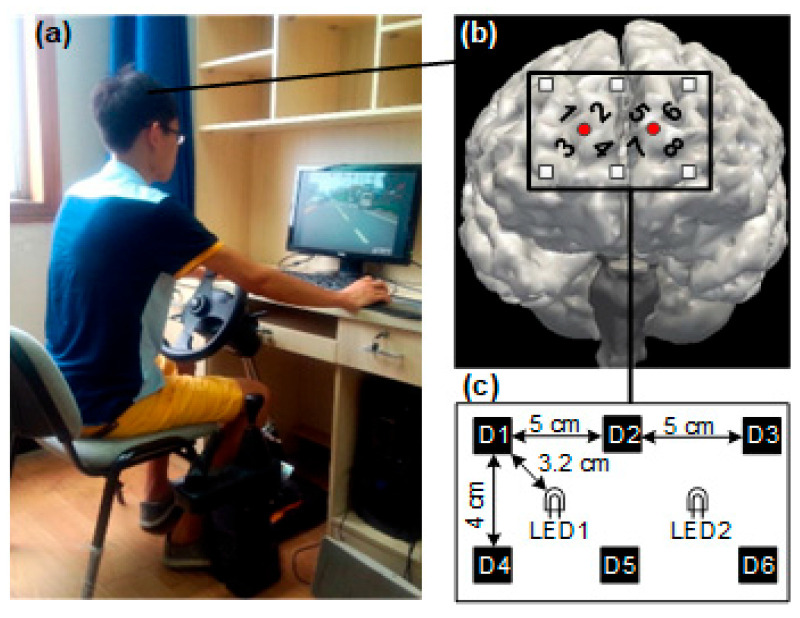
The arrangement of light sources and detectors. (**a**) Experiment environment; (**b**) channel location; (**c**) device design.

Flexible film probes were employed in this study, covering the foreheads of participants (Figure 3). These probes were equipped with two continuous-wave (CW) LED light sources at wavelengths of 735/805/850 nm and six near-infrared light sensors. Positioned around the light sources, the sensors formed a rectangle measuring 4 cm × 10 cm, encompassing the area between the forehead, eyebrows, and hairline. The distances between LED1 to D3 and D6, and LED2 to D1 and D4 were too extensive to yield reliable near-infrared spectroscopy data. Consequently, data from these channels were excluded from analysis. Subsequently, eight source–detector (SD) pairs, or fNIRS signal channels, were established. These pairs maintained a consistent distance of 3.2 cm and enabled penetration depths of >2 cm from the skin to brain tissue. The two light sources operated alternately, with detailed channel positions depicted in Figure 3b.

In this investigation, the alteration in the level of oxygenated hemoglobin concentration (Δ[HbO_2_]) in comparison to the start of the fNIRS data recording is represented by Δ[HbO_2_], whereas the change in the level of deoxygenated hemoglobin concentration (Δ[Hb]) relative to the initiation of the fNIRS data recording is indicated by Δ[Hb]. Furthermore, Δ[tHb] denotes the modification in the level of total hemoglobin concentration compared to the state of the fNIRS data recording. By applying the modified Beer–Lambert law, alterations in the concentrations of oxyhemoglobin (Δ[HbO_2_]) and deoxyhemoglobin (Δ[Hb]) were calculated based on light intensity The total hemoglobin (Δ[tHb], Δ[tHb] = Δ[Hb] + Δ[HbO_2_]) was obtained by Δ[Hb] + Δ[HbO_2_].

### 2.3. Data Process and Analysis

This study collected behavioral data from non-voluntary attention brain function experiments, including task accuracy and reaction time (RT). A composite parameter that simultaneously responds to both parameters was derived by calculating accuracy/RT [25]. The data were fitted using Fourier second-order analytical equations, fourth-order polynomial equations, and sine analytical equations. The goodness of fit (R-square) and sum of squared errors (SSE) between the actual data and corresponding predicted values were used to evaluate the performance of the three fitting methods. For each participant, each method yielded an R-square and an SSE, resulting in eight R-squares and eight SSEs for each method. Subsequently, one-way analysis of variance (ANOVA) was conducted to determine whether there were significant differences in R and SSE among the three methods. The optimal method was selected for fitting, and the slope of the fitted curve was analyzed to obtain trends in behavioral performance changes.

Applying the modified Beer–Lambert law to fNIRS data enables the derivation of Δ[HbO_2_], Δ[Hb], and Δ[tHb] for eight distinct channels. These datasets facilitate the visualization of alterations in prefrontal hemodynamic parameters throughout the driving task. This analysis permits the identification of the channel exhibiting the most substantial fluctuations in hemodynamic parameters over time, thus allowing for subsequent correlation analyses with behavioral metrics. By examining the association between individual hemodynamic parameters and behavioral metrics, one can discern the parameters most responsive to variations in behavioral data.

## 3. Results

### 3.1. Behavior Result

By monitoring task accuracy and RT associated with visual involuntary attention, participants’ performance can be tracked, and these metrics are subsequently utilized to compute accuracy/RT ratios.

Given that the task accuracy and RT data collected in this experiment consist of discrete data points, a mathematical model was constructed to analyze the data trend. Three equations were employed for this purpose: the Fourier second-order analytical equation (Equation (1)), fourth-order polynomial equation (Equation (2)), and sinusoidal analytical equation (Equation (3)). These equations, denoted by various parameters such as *a*_0_, *a*_1_, *a*_2_, *b*_0_, *b*_1_, and *w* (for Fourier), *p*_0_, *p*_1_, *p*_2_, *p*_4_, and *p*_5_ (for polynomial), and *a*, *b*, and *c* (for sinusoidal), were fitted to the data using variable *x*.

(1) $$f(x)=a_{0}+a_{1}\cos(xw)+b_{1}\sin(xw)+a_{2}\cos(2xw)+b_{2}\sin(2xw)$$

(2) $$f(x)=p_{1}x^{4}+p_{2}x^{3}+p_{3}x^{2}+p_{4}x+p_{5}$$

(3) f(x)=asin(bx+c)

The R-square and SSE analyses indicated that each dataset adhered to a normal distribution, as confirmed by the Shapiro–Wilk normality test. Specifically, the Fourier second-order analytical equation exhibited an R-square (*p* = 0.748, n = 7) and SSE (*p* = 0.891, n = 7), the fourth-order polynomial equation showcased an R-square (*p* = 0.943, n = 7) and SSE (*p* = 0.886, n = 7), while the sinusoidal analytical equation displayed an R-square (*p* = 0.868, n = 7) and SSE (*p* = 0.917, n = 7). Subsequent one-way ANOVA revealed significant differences in R-square among the three models (F = 64.693, *p* < 0.001, partial η^2^ = 0.902).

Post hoc comparisons demonstrated that both the Fourier second-order analytical equation and the fourth-order polynomial equation significantly outperformed the sinusoidal analytical equation (Fourier: t = 9.791, *p* < 0.001, Cohen’s d = 3.638; fourth-order polynomial: t = 9.909, *p* < 0.001, Cohen’s d = 3.682) in terms of R-square. Moreover, the SSE analysis revealed significant differences among the three models (F = 29.704, *p* < 0.001, partial η^2^ = 0.809), with both the Fourier and fourth-order polynomial equations yielding significantly lower SSE values than the sinusoidal equation (Fourier: t = −6.682, *p* < 0.001, Cohen’s d = −2.032; fourth-order polynomial: t = −6.668, *p* < 0.001, Cohen’s d = −2.028).

Although no significant disparity was observed between the R-square and SSE of the Fourier second-order analytical equation and the fourth-order polynomial equation, the SSE of the former (mean = 0.052 × 10^−6^) was marginally lower than that of the latter (mean = 0.0526 × 10^−6^). Therefore, subsequent analyses were conducted using the Fourier second-order analytical equation for data fitting.

**Figure 4:**
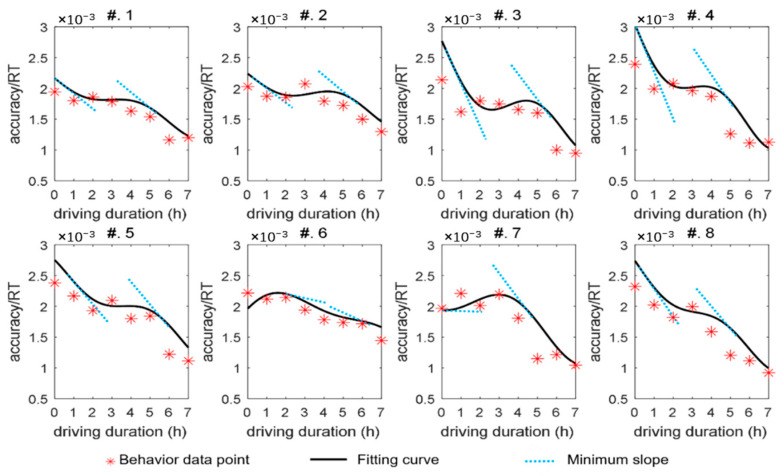
Changes in accuracy/RT ratio for 8 participants.

The results of the fitting are depicted in Figure 4. By integrating the preceding analysis of discrete data, the entire 7 h driving session can be segmented into three phases, comprising two rapid decline phases and one gradual decline phase. Upon commencing the driving simulation, the majority of participants experienced a continuous decrease in behavioral performance until the 2 h mark, followed by a period of stability lasting approximately 2 h, after which another decline ensued. The steepness of the fitting curve’s slope illustrates the trend in performance variation. As delineated in these findings, it becomes evident that, for most participants, the slope during the second rapid decline phase was steeper than that observed during the first phase. By the 4 h mark of the driving session, a significant deterioration in driving performance was observed among most drivers.

### 3.2. fNIRS Results

**Figure 5:**
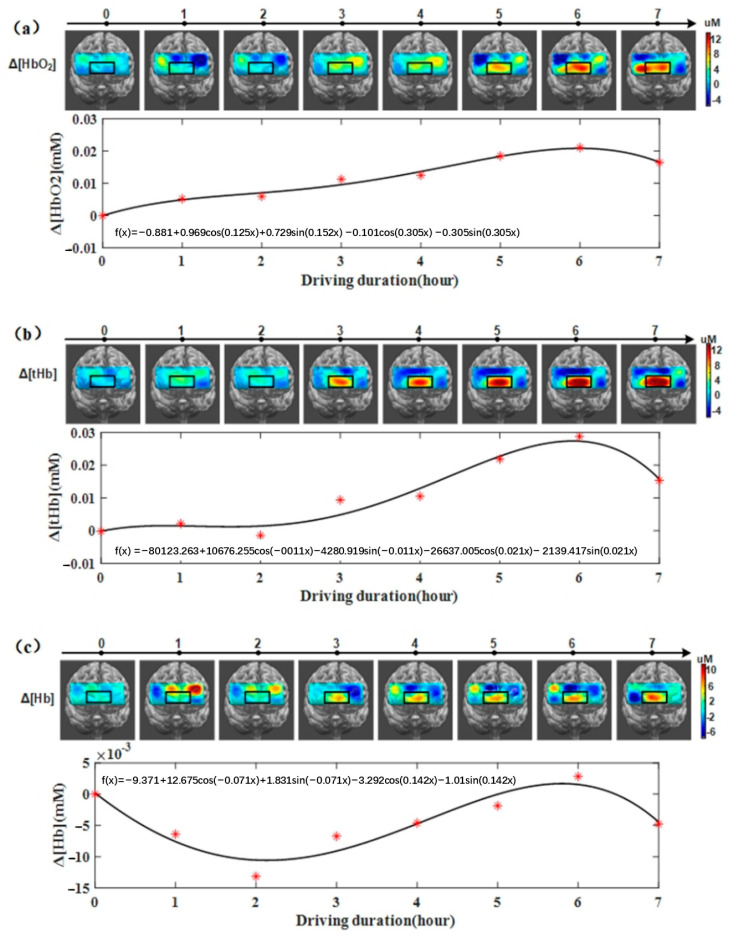
The mean results of the prefrontal activation map and change in concentration of channel 7, interpreted by the three hemodynamic parameters: (**a**) the results of Δ[HbO_2_]; (**b**) the results of Δ[tHb]; (**c**) the results of Δ[Hb].

Prefrontal activation mapping was derived from the hemodynamic data collected from eight channels positioned on the forehead. Figure 5 illustrates the average Δ[HbO_2_], Δ[tHb], and Δ[Hb] values for eight participants, with the region exhibiting the most pronounced changes in hemodynamic parameters highlighted by a black rectangle. It is evident from Figure 5 that as the experimental duration progresses, there is a noticeable increase in Δ[HbO_2_], Δ[tHb], and Δ[Hb] within this highlighted area. Notably, Δ[tHb] exhibited the highest sensitivity (maximum value) to the experimental duration. Moreover, significant alterations in activation patterns were observed during the initial 2 h in Δ[HbO_2_], Δ[tHb], and Δ[Hb]. Throughout the experiment, there was marked spatial variation in activation patterns during the first 2 h, which gradually diminished as the driving duration reached 4 h, accompanied by a continuous rise in the concentrations of Δ[HbO_2_], Δ[tHb], and Δ[Hb] within the highlighted region.

During most of the experimental process, channel 7 demonstrated the most pronounced activation. Table 1 presents the concentration changes of Δ[HbO_2_], Δ[tHb], and Δ[Hb] for channel 7. It is apparent that, for the majority of participants, Δ[HbO_2_] and Δ[tHb] for channel 7 exhibited an upward trend during prolonged driving, while Δ[Hb] for channel 7 displayed some fluctuations with a sinusoidal pattern. Spearman correlation coefficients were then computed between accuracy/RT and the hemodynamic parameters of channel 7 (Table 2). Significant negative correlations were observed between Δ[HbO_2_] and accuracy/RT in seven out of eight participants. Additionally, four participants exhibited a significant negative correlation between their Δ[tHb] data and accuracy/RT, while three participants demonstrated a significant negative correlation between their Δ[Hb] data and accuracy/RT.

## 4. Discussion

To explore the impact of prolonged driving on both performance and brain function, we conducted a visual involuntary brain function experiment to evaluate changes in visual involuntary attention, as indicated by accuracy/RT, throughout a seven-hour driving simulation. Simultaneously, fNIRS was employed to capture hemodynamic alterations during the experimental session. The findings reveal a distinctive nonlinear decline in accuracy/RT values over the course of the seven-hour experiment, delineated into two rapid decrease phases and one gradual decrease phase based on the fitting outcomes. The hemodynamic findings illustrate an upward trend in Δ[HbO_2_] and Δ[tHb], evident in the activation area and signals obtained from channel 7. Furthermore, a robust negative association was noted between Δ[HbO_2_] and accuracy/RT among the majority of participants (seven out of eight).

In earlier investigations, scholars primarily focused on the initial three hours of fatigued driving, which typically revealed only a single rapid decline phase. Ting et al., for instance, conducted a two-hour driving test and proposed that 80 min represented the maximum safe driving duration for highway driving, as drivers reported experiencing fatigue beyond this timeframe [26]. Nilsson et al. reached a comparable conclusion [27]. Nevertheless, the restricted duration of experiments in these studies may have led to potentially misleading findings. In our research, we extended the driving test to 7 h, allowing for an examination of behavioral changes beyond the initial three hours. Our analysis of behavioral data unveiled two rapid decline phases and one gradual decline phase during the experiment. Performance deterioration commenced within the first 2 h, followed by a period of relative stability lasting approximately 2 h, after which a substantial decline in performance ensued.

Our research findings indicate that participants’ performance varies across different stages. The study involved a total of eight participants (five males and three females), with six participants demonstrating performance across three stages, and two participants (one male and one female) across two stages. Regardless of the number of stages, there is always a rapid decline stage, beginning around 4 h, where participants’ performance sharply decreases. According to current national standards, drivers should take a rest after driving continuously for 4 h, which aligns with our research results. It is worth emphasizing that our study utilized only eight participants, yet the results suggest that combining fNIRS with non-voluntary attention brain function experiments allows for relatively precise conclusions to be drawn with a relatively small sample size.

Combining Figure 5 with Figure 4, it can be observed that the rapid decline phase in RT/accuracy aligns with the rapid accumulation phase of ΔHBO_2_, both occurring around 4 h. This indicates consistency between the behavioral and hemodynamic results obtained in this study. Therefore, 4 h appears to be a crucial time point for both behavioral and hemodynamic outcomes. Additionally, the strong negative correlation between Δ[HbO_2_] of the seventh channel and the behavioral results of most participants (seven out of eight) supports the feasibility of establishing a fatigue monitoring system through fNIRS and non-voluntary attention brain function experiments.

Due to the limited scope of the scenarios used in the study and the dataset only including eight people, the results presented in this paper are preliminary. Therefore, future research should focus on expanding the dataset to ensure the validity of the results and conclusions. Additionally, as the experiments were conducted in a driving simulation environment, it is necessary to test the current research results in more advanced driving simulators to confirm the consistency of the conclusions and maintain participant safety.

Furthermore, it is necessary to identify user-friendly electroencephalogram (EEG) acquisition methods suitable for long-term data collection to monitor brain activity during prolonged driving, in conjunction with near-infrared spectroscopy (NIRS). The methodology proposed in this study can be integrated with other research topics, such as investigating intervention measures to alleviate fatigue and reduce its impact. Previous studies have investigated methods such as music [28], odors [29], and temperature [30] to mitigate fatigue. Therefore, combining the approach outlined in this paper with fatigue mitigation techniques holds great promise.

## 5. Conclusions

In conclusion, this study proposes a novel approach to assess prolonged fatigue. fNIRS data analysis reveals a significant negative correlation between behavioral data and Δ[HbO_2_], indicating a strong association between driver performance and hemodynamic changes. Based on behavioral outcomes, it is suggested that there is always a rapid phase, starting at approximately 4 h, leading to a sharp decline in performance. According to current national standards, drivers should take a break after driving continuously for 4 h, which aligns with our research findings. The conclusion drawn in this paper, based on the combination of fNIRS and non-voluntary attention brain function experiments, was derived from a sample size of only eight participants, demonstrating the potential application value of this method in the field of prolonged fatigue detection.

## Figures and Tables

**Table 1:** The concentration changes of Δ[HbO_2_], Δ[tHb], and Δ[Hb] for channel 7 (mean ± std).

Time Length	Δ[HbO_2_] (mM)	Δ[tHb] (mM)	Δ[Hb] (mM)
0 h	−0.0001 ± 0.001	−0.0002 ± 0.0013	0 ± 0.0011
1 h	0.0052 ± 0.0061	0.0022 ± 0.0129	−0.0064 ± 0.0206
2 h	0.0059 ± 0.0081	−0.0015 ± 0.0192	−0.0132 ± 0.0310
3 h	0.0113 ± 0.0070	0.0094 ± 0.0229	−0.0067 ± 0.3410
4 h	0.0125 ± 0.0077	0.0105 ± 0.0146	−0.0046 ± 0.0248
5 h	0.0185 ± 0.0060	0.0219 ± 0.0281	−0.0019 ± 0.0359
6 h	0.0211 ± 0.0072	0.0287 ± 0.0408	0.0028 ± 0.0460
7 h	0.0164 ± 0.0087	0.0153 ± 0.0259	−0.0048 ± 0.0364

**Table 2:** Correlation coefficient between accuracy/RT and Δ[HbO_2_], Δ[tHb], and Δ[Hb] of channel 7.

Participant	Δ[HbO_2_]		Δ[tHb]		Δ[Hb]	
	rho	*p*	rho	*p*	rho	*p*
#.1	−0.95	<0.01 **	−0.95	<0.01 **	−0.95	<0.01 **
#.2	−0.31	0.46	−0.21	0.61	−0.24	0.58
#.3	−0.74	<0.05 *	−0.88	<0.01 **	−0.9	<0.01 **
#.4	−0.9	<0.01 **	−0.98	<0.01 **	−0.95	<0.01 **
#.5	−0.81	<0.05 *	−0.86	<0.05 *	0.1	0.84
#.6	−1	<0.001 **	−1	<0.001 ***	−0.62	0.11
#.7	−0.74	<0.05 *	−0.12	0.79	0.45	0.27
#.8	−0.9	<0.01 **	0.12	0.79	0.6	0.13

Note: * represents significant difference (* *p* < 0.05; ** *p* < 0.01; *** *p* < 0.001).

## Data Availability

The data involved in this study are not disclosed.
